# Lactobacillli expressing llama VHH fragments neutralise *Lactococcus *phages

**DOI:** 10.1186/1472-6750-7-58

**Published:** 2007-09-17

**Authors:** Anna Hultberg, Denise M Tremblay, Hans de Haard, Theo Verrips, Sylvain Moineau, Lennart Hammarström, Harold Marcotte

**Affiliations:** 1 Division of Clinical Immunology at the Department of Laboratory Medicine, Karolinska Institutet at Karolinska University Hospital in Huddinge, Stockholm, Sweden; 2 Groupe de recherche en écologie buccale, Faculté de médecine dentaire, Félix d'Hérelle Reference Center for Bacterial Viruses, Université Laval, Québec, G1K 7P4, Canada; 3 Unilever Research and Development, Vlaardingen, The Netherlands; 4 Cellular Architecture and Dynamics (CAD), Utrecht University, Utrecht, The Netherlands; 5 Département de biochimie et de microbiologie, Faculté des sciences et de génie, Université Laval, Québec, G1K 7P4, Canada; 6 Ablynx, Technologiepark 4, 9052 Ghent, Belgium; 7 Cellular Architecture and Dynamics (CAD), Utrecht University, The Netherlands

## Abstract

**Background:**

Bacteriophages infecting lactic acid bacteria (LAB) are widely acknowledged as the main cause of milk fermentation failures. In this study, we describe the surface-expression as well as the secretion of two functional llama heavy-chain antibody fragments, one binding to the major capsid protein (MCP) and the other to the receptor-binding proteins (RBP) of the lactococcal bacteriophage p2, by lactobacilli in order to neutralise lactococcal phages.

**Results:**

The antibody fragment VHH5 that is directed against the RBP, was fused to a c-*myc *tag and expressed in a secreted form by a *Lactobacillus *strain. The fragment VHH2 that is binding to the MCP, was fused to an E-tag and anchored on the surface of the lactobacilli. Surface expression of VHH2 was confirmed by flow cytometry using an anti-E-tag antibody. Efficient binding of both the VHH2 and the secreted VHH5 fragment to the phage antigens was shown in ELISA. Scanning electron microscopy showed that lactobacilli expressing VHH2 anchored at their surface were able to bind lactococcal phages. A neutralisation assay also confirmed that the secreted VHH5 and the anchored VHH2 fragments prevented the adsorption of lactococcal phages to their host cells.

**Conclusion:**

Lactobacilli were able to express functional VHH fragments in both a secreted and a cell surface form and reduced phage infection of lactococcal cells. Lactobacilli expressing llama heavy-chain antibody fragments represent a novel way to limit phage infection.

## Background

Llamas, a member of the *Camelidae *family, produce heavy chain antibodies, a type of antibodies that lack the CH1 domain and light chains [1]. The antigen binding portion of these antibodies, called VHH, can be expressed at high levels in *Saccharomyces cerevisiae *[2]. VHH antibody fragments have already shown a considerable potential in several biotechnological applications such as decreasing the amount of smooth surface caries in a rat model [3], shortening disease duration, severity and viral load in a mouse model of rotavirus-induced diarrhea [4], and preventing phage infection of *Lactococcus *cells during milk fermentation [5,6].

Virulent bacteriophages infecting lactic acid bacteria (LAB) are widely acknowledged as the main cause of milk fermentation failures and they are also responsible for the downgrade of fermented dairy products such as cheeses [7,8]. Their ubiquity in dairy environments, biodiversity, and genomic plasticity are largely responsible for the difficulty in controlling phage infection [9,10]. Consequently, several tactics have been proposed to curtail their proliferation in industrial settings [10]. The generation of phage neutralising VHH antibodies is one of the latest antiviral strategies that have been proposed to inhibit lactococcal phages [5,6]. As a proof of concept, a panel of non-neutralising and neutralising VHH antibody fragments targeting the lactococcal isometric-headed 936-type phage p2, was recently obtained [5]. The direct addition of one of them (VHH5) to milk prevented the infection of the strain *Lactococus lactis *subsp. *cremoris *C2 by the virulent phage p2 during the manufacture of a Gouda-type cheese [6]. The VHH5 fragment effectively inhibited lactococcal phage infection by directly binding to the receptor-binding protein (RBP/ORF18) located at the distal part of the phage tail [5]. Recently, it was shown that other phages belonging to the predominant lactococcal 936 species, could also be neutralised by this antibody [11]. Moreover, some of the non-neutralising fragments, such as VHH2, were shown to bind to the major structural capsid protein (ORF11) of phage p2 [5].

Lactobacilli are also Gram-positive lactic acid bacteria that normally colonize the oro-gastrointestinal tract [12,13]. Some *Lactobacillus *strains are believed to have health promoting properties and are used as supplements in dairy products, either alone or in combination with other microorganisms [14,15]. Similarly to *Lactococcus lactis *strains, other carefully selected *Lactobacillus *strains are an integral part of industrial starter cultures that are added to milk for the manufacture of an array of fermented dairy products. Thus, their large-scale used in the food industry is well established and their long history of safe use has led to their status as a Generally Regarded As Safe (GRAS) microorganism. This GRAS status has led to reports in which lactobacilli were suggested as carriers for passive immunization through surface expression or secretion of various antibodies [16]. Recently, functional antibody fragments targeting pathogenic bacteria (*Streptococcus mutans *and *Porphyromonas gingivalis*) and a human virus (rotavirus) have been produced in lactobacilli [4,16-18] and shown to have an antimicrobial potential.

In this study, we have explored the possibility of producing functional VHH antibody fragments by lactobacilli in order to neutralise lactococcal phages. The *in situ *VHH production, in a secreted form or anchored to the cell surface, could potentially alleviate the need to add the VHH fragments directly to the fermentation medium, thereby reducing the costs of the technology.

## Results and discussion

### Construction of *Lactobacillus paracasei *strains expressing VHH fragments that bind to structural proteins of *Lactococcus lactis *phage p2

**Figure 1:**
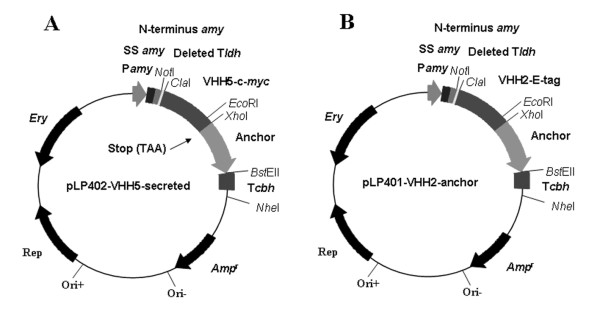
**Map of the *Lactobacillus *expression vectors**. (A) pLP402-VHH5-secreted with a stop codon (TAA) inserted after the E-tag sequence, mediating secretion of the antibody fragment and (B) pLP401-VHH2-anchored mediating surface-anchored expression of antibody fragments by fusion to the last 244 amino acids of the *Lactobacillus casei *proteinase P. P*amy*, Promotor sequence of the α-amylase gene of *L. amylovorus*; SS *amy*, signal sequence of the α-amylase gene of *Lactobacillus amylovorus *(36 amino acids); N-terminus *amy*, N-terminus (26 amino acids) of the α-amylase of *L. amylovorus*; deleted T*ldh*, remaining sequence after deletion of transcription terminator of the lactate dehydrogenase gene of *L. casei*; VHH5, VHH fragment against the receptor-binding protein of lactococcal phage p2; VHH2, VHH fragment against the major structural capsid protein of phage p2; Anchor, anchor sequence from the proteinase P gene of *L. casei *(244 amino acids); T*cbh*, transcription terminator sequence of the conjugated bile acid hydrolase gene of *Lactobacillus plantarum *80; *Amp*^r^, ampicillin-resistance gene; *Ery*, erythromycin-resistance gene.

We expressed the VHH5 single-chain as a secreted product and the VHH2 as a surface anchored fusion protein in *L. paracasei*. This strategy was selected because it was previously shown that the addition of VHH5 to a culture medium prevented phage infection while VHH2 had no effect [5]. However, we reasoned that anchoring VHH2 to a cell surface might prevent phage infection by tittering out viral particles present in a medium. First, the VHH2-E-tag and VHH5-c-*myc *fragments were inserted into the *Lactobacillus *expression vector pLP401, respectively generating the vectors pLP402-VHH5-secreted and pLP401-VHH2-anchored (Fig. 1A and 1B).

The presence of pLP402-VHH5-secreted in *L. paracasei *was found to mediate the secretion of VHH5 into the medium (Fig 1A). The introduction of pLP401-VHH2-anchored into *L. paracasei *led to the cell surface expression of VHH2 by fusion to the C-terminal cell wall anchored domain of proteinase P (Fig. 1B). In these vectors, the VHH expression is under the control of the promoter of the *amy *gene (Fig. 1). This regulatable promoter is repressed by sugars transported by the phosphoenolpyruvate-dependent phosphotransferase systems (PTS). De-repression of the promoter and protein expression is obtained by growing the cells in the presence of non-PTS sugars such as mannitol. Both VHH fragments were tagged for detection with anti-E-tag or anti-myc-tag antibodies. The theoretical molecular mass of the anchored VHH2 and secreted VHH5 is 43 kDa and 23 kDa respectively after cleavage of the signal peptide (4 kDa) but containing the N-terminus (26 amino acids, 3.9 kDa) of the amylase protein fused to the VHH (Fig. 1).

### Expression of the VHH fragments

**Figure 2:**
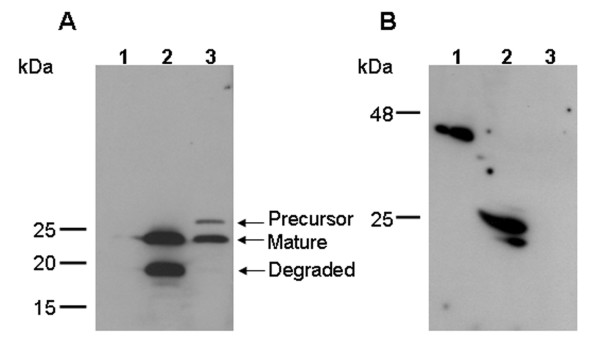
**Determination of the expression of VHH fragments by *L. paracasei *using Western blot assay**. (A) lane 1, supernatant of pLP402; lane 2, supernatant of pLP402-VHH5-secreted showing the secreted mature VHH of 23 kDa and a degradation product of 19 kDa; lane 3, cell extracts of pLP402-VHH5-secreted showing the precursor VHH (signal peptide-VHH) of 26 kDa and the mature VHH of 23 kDa. (B) lane 1, cell extracts of pLP401-VHH2-anchored showing a anchored VHH2 of 46 kDa; lane 2, control VHH2-E-tag (supernatant from pLP402-VHH2-secreted); lane 3; cell extracts of pLP402.

VHH expression was analysed by immunoblotting of *L. paracasei*-transformed strains using monoclonal anti-E-tag antibodies or anti-myc antibodies. The culture supernatant and cell pellet of *L. paracasei *transformed with the pLP402-VHH5-secreted vector contained a protein of 23 kDa corresponding to the secreted mature VHH (Fig. 2A). An additional band of approx. 19 kDa in the supernatant of *L. paracasei *transformed with the pLP402-VHH5-secreted could also be observed, probably resulting from degradation. In all likelihood, the degradation did not occur in the binding part of the VHH but rather in the N-terminal part of the amylase protein fused to the VHH after cleavage of the signal peptide (Fig. 1).

**Figure 3:**
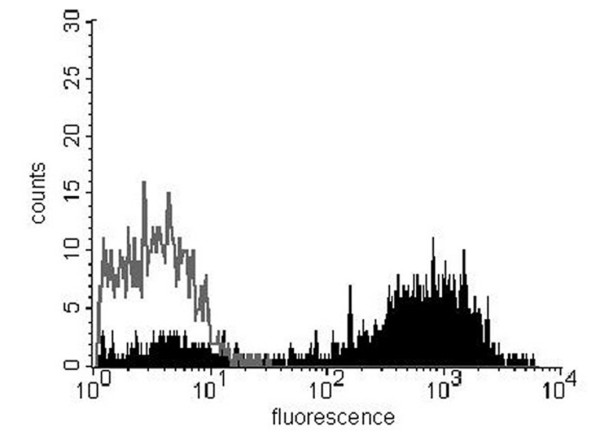
**Flow cytometry showing VHH2 expression on the surface of L. paracasei**. *L. paracasei *transformed with the pLP401-VHH2-anchored (black filled). The negative control, *L. paracasei *containing the vector pLP402, did not show any signal (grey line).

The cell extract of *L. paracasei *transformed with pLP401-VHH2-anchored yielded a protein of approximatively 46 kDa (Fig. 2B). Surface expression of VHH2 on recombinant lactobacilli transformed with pLP401-VHH2-anchored was analysed by flow cytometry using an anti-E-tag antibody. The transformed pLP401-VHH2-anchored *L. paracasei *strain showed a strong positive signal when stained with the anti-E-tag antibody, whereas the same parental strain transformed with the vector pLP402 did not show any signal (Fig. 3). *L. paracasei *transformed with pLP402-VHH5-secreted did not show any surface expression in flow cytometry (data not shown). Taken altogether, these findings confirm the surface location of the VHH2-E-tag-anchored fusion construct on the cell.

Using purified VHH5 and VHH2 fragments as positive controls, we also estimated that the *L. paracasei *strain containing the vector pLP401-VHH2-anchored expressed about 10^3^–10^4 ^molecules per cell (calculated from the VHH standard) while the *L. paracasei *strain carrying pLP402-VHH5-secreted, grown to an OD_600 _of 0.8, expressed about 500 ng of VHH fragments per ml of supernatant, corresponding to a production rate of roughly 5 × 10^4 ^VHH fragments/bacterium/hour.

### Binding of the expressed VHH fragments to phage p2

**Figure 4:**
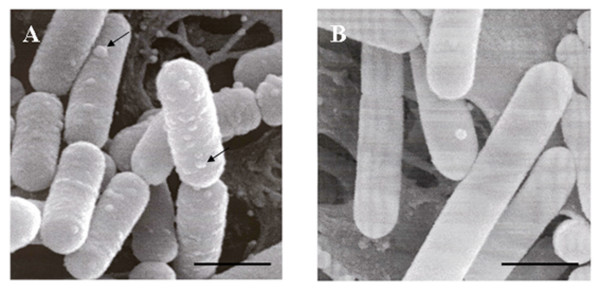
**Scanning electron microscope (SEM) image**. (A) Phage p2 particles binding to the recombinant *L. paracasei *expressing VHH2 anchored on the cell surface (approximately 20 times more binding than the control). (B) Control, *L. paracasei *containing only the vector pLP402 mixed with lactococcal phage p2. Arrows indicate the phage p2 particles. Bar = 1 μm.

Binding of the VHH fragments to their respective antigen was analysed by ELISA using homogenates of the *L. paracasei *pLP401-VHH2-anchored and supernatant from *L. paracasei *pLP402-VHH5-secreted. Both VHH fragments were able to bind to their antigen indicating that they are functional (Table 1). No activity was observed using extracts and supernatants from cultures of *L. paracasei *containing only the pLP402 vector. The *Lactobacillus *strain expressing the anchored VHH2 was also mixed with *Lactococcus *phage p2 and analyzed by scanning electron microscopy (SEM). Phage particles were found at the surface of the recombinant lactobacilli expressing the anchored VHH2 but not at the surface of *L. paracasei *containing only the pLP402 vector (Fig. 4).

**Table 1:** Binding of the expressed VHH fragments to phage p2 antigens by ELISA

	Supernatant^a^		Cell extract^b^	
Dilution^c^	pLP402-VHH5-secreted	pLP402	pLP402-VHH2-anchored	pLP402

undiluted	0.987	0.076	1.549	0.084
1/4	0.519	-	1.207	-
1/16	0.339	-	0.511	-
1/64	0.128	-	0.215	-

^a ^Supernatant from a culture grown until an OD_600 _nm of 0.8.

^b ^Cell extract was made by sonication of 2.5 × 10^9 ^CFU in 1 ml of PBS.

^c ^Undiluted or dilutions of supernatant and cell extract were added to the wells.

### Neutralisation of phage p2 by VHH2 and VHH5 expressed in lactobacilli

Finally, the ability of recombinant *Lactobacillus *strains to neutralise p2 phages was also studied. A fixed titer of phage (1,000 pfu) was mixed with supernatant or *Lactobacillus *cells expressing VHH. The supernatant from *Lactobacillus *pLP402-VHH5-secreted was shown to neutralise phage p2 by binding to its RBP and significantly inhibiting (86%) its adsorption to the host strain *Lactococcus lactis *MG1363. In comparison, almost no p2 phage particles were bound to the parental *L. paracasei *strain with or without the cloning vector pLP402 (Table 2). Interestingly, the surface expressed VHH2 was also able to inhibit phage infection of *L. lactis *cells, albeit to a lesser extent (31%) (Table 2). Thus, even though VHH2 (which is recognizing the major capsid protein) is non-neutralising when added as a monovalent fragment to a culture medium [5,19], it can neutralise bacterial viruses when anchored at the surface of a lactic acid bacterium. As compared to other anti-phage strategies currently available, the above reported inhibitory effect of VHH (as a secreted or anchored product) would probably not be considered as a strong anti-phage system (7). However, lactic acid bacteria producing VHH could be used concomitantly with other strategies to increase the overall protection of starter cultures against phages, thereby providing a novel hurdle to control them.

**Table 2:** Percentage of phage p2 inhibition by bacterially produced VHH

Bacteria	% inactivation^a^	
	
	Supernatant^b^	Bacteria^c^
*L. paracasei *parental	1.8 ± 5.9	2.1 ± 1.0
*L. paracasei *+ pLP402	4.6 ± 4.0	-4.4 ± 6.4
*L. paracasei *+ pLP402-VHH5-secreted	86.0 ± 5.1	ND
*L. paracasei *+ pLP402-VHH2-anchored	3.4 ± 6.6	31.4 ± 2.8

^a ^Mean ± standard deviation of at least three experiments

^b ^10 μl supernatant (approximately 5 ng VHH)

^c ^4 × 10^6 ^bacteria

## Conclusion

It was previously shown that VHH5 fragments bind to some but not all 936-like phages [11], which is the most prevalent lactococcal phage group in the dairy industry [5,9]. Moreover, it was recently shown that phage mutants no longer neutralised by VHH5 could be readily isolated in the laboratory [11]. Further strategies are thus needed to improve the broadness of the VHH protection and to prevent the emergence of new virulent phages. One possible approach could be the identification of more potent neutralising VHH fragments. Alternatively, the expression of multiple VHH fragments could enhance the protection and the applicability of the system. It should be noted that the VHH fragment should preferably be constitutively produced using an expression system devoid of antibiotic selection marker. Nonetheless, the proof of concept reported here univocally showed that the expression of anti-phage VHH by a LAB represents a novel tool to prevent phage infection. This method would be much more cost effective as the VHH fragments would be produced *in situ *in the fermentation medium, eliminating the need for additional purification of the VHH fragments.

## Methods

### Bacterial strains, phage, culturing conditions, and VHH expression

*Escherichia coli *DH5α was used as the cloning host strain and cells were grown in Luria-Bertani (LB) medium (10 g tryptone/litre, 5 g NaCl/litre, 5 g yeast extract/litre). *E. coli *transformants were selected on LB plates containing 100 μg/ml ampicillin. *Lactobacillus paracasei *(previously known as *L. casei *or *L. zeae *ATCC 393 pLZ15^-^) [20], transformed with the plasmids pLP402 [16], pLP402-VHH5-secreted or pLP401-VHH2-anchored, were selected on MRS (Difco) plates with 3 μg/ml erythromycin after cultivation anaerobically at 37°C for 48 h. The pLP402-VHH5-secreted and pLP401-VHH2-anchored vectors, respectively mediated the secretion of VHH5 fragments and the surface expression of VHH2 fragments under the transcriptional control of the regulatable α-amylase promoter. The α-amylase promoter is regulated by a negative feedback. It is repressed by PTS sugars such as glucose and lactose in *L. paracasei*. Growth in presence of non-PTS sugars, such as mannitol, derepresses the promoter and activate gene expression. Pre-cultures of *L. paracasei *were made by growing the cultures in LCM medium [21] supplemented with 1% glucose and 3 μg/ml of erythromycin when needed and incubating them at 37°C overnight. These cultures were used to inoculate (2%) LCM-Man medium supplemented with 0.5% mannitol and 3 μg/ml of erythromycin, which were then incubated at 37°C. Cells were harvested in the exponential growth phase at an optical density at 600 nm (OD_600_) of 0.8 (10^8 ^cfu/ml) [16,21]. *Lactococcus lactis *MG1363 was grown at 30°C in M17 broth [22] supplemented with 0.5% glucose (GM17) (Difco). Lysate of the lactococcal phage p2 was prepared as described previously [23].

### Construction of the Lactobacillus paracasei expression vectors pLP401-VHH2-anchor and pLP402-VHH5-secreted

The VHH2 encoding gene was cut out from the phagemid vector pUR3824 [5] at the restriction sites *Sfi*I and *Not*I and ligated into pCANTAB 5E (Amersham Pharmacia Biotech) in order to fuse it with the E-tag. PCR amplification of the VHH2-E-tag was performed to add restriction sites for *Cla*I and *Xho*I to the VHH2-E-tag using primers *Cla*I-VHH: 5'-GCCATTGGAACTTACTCTGAAAA-3' and *Xho*I-VHH: 5'-CCGCTCGAGTGCGGCACGCGGTTCC-3'. Similarly, the VHH5-c-*myc *fragment was amplified by PCR from the pUR3825 [5] vector using the primers *Cla*I-VHH and c-*myc*-stop (5'-CCGCTCGAGTTATGCGGCACGCGGTTCC-3') and adding in the process the restriction sites *Cla*I and *Xho*I with a stop codon (TAA) after the c-*myc *gene. The VHH2-E-tag and VHH5-c-*myc *fragments were, after restriction cutting and purification, ligated into the *Lactobacillus *expression vector pLP401 (previously named pLP402) [16,21] at the *Cla*I and *Xho*I sites to generate the vectors pLP401-VHH2-anchored and pLP402-VHH5-secreted. Transformation of *L. paracasei *was performed as previously described [16,21]. Selection of positive clones was performed using MRS plates containing 3 μg/ml erythromycin. Lactobacilli containing pLP402-VHH2-secreted was also constructed, similarly to the pLP401-VHH2-anchored construct but with a stop (TAA) before the E- tag and was used as control in the Western blot assays.

### Preparation of samples for the enzyme-linked immunosorbent assay and Western blot analysis

After growth in presence of 0.5% mannitol, cells were washed, treated with lysosyme and disrupted by sonication as previously described [17]. For ELISA, cell debris were removed by centrifugation for 10 min at 10,000 × *g *and the supernatant containing the protein extracts were stored at -20°C before use. For the Western blot analyses, Laemmli loading buffer was added to the sonicated cell extracts and the samples were boiled for 5 min, centrifuged (15 min, 10,000 × *g*) after which the supernatants were stored at -20°C. Supernatants from cultures of *L. paracasei *secreting VHH5 were filtered (0.45 μm) and concentrated 50 times using an ultrafiltration unit (Amicon). Protein concentrations were determined by the BioRad protein assay (BioRad Laboratories). For Western blot, the concentrated supernatant was boiled for 5 min in Laemmli buffer.

### Flow cytometry

After growth in presence of mannitol, 100 μl of each lactobacilli culture (10^7 ^bacteria) containing the vectors pLP402 or pLP401-VHH2-anchored were washed three times in PBS by centrifugation (10,000 × *g *for 15 min) before resuspension in 100 μl of PBS. An equal amount of mouse anti-E-tag antibody (Amersham Bioscience) diluted 1/200 was added and the samples were incubated on ice for 1 h. The washing procedure in PBS was repeated and the samples were resuspended in 100 μl of PBS and mixed with 100 μl cy2-labeled donkey anti-mouse antibodies (Jackson Immunoresearch Laboratories) (final dilution 1/200) and incubated on ice for 30 min. After washing, the samples were resuspended in one ml of PBS and analysed in a FACSCalibur machine (Becton Dickinson).

### Western blotting

Samples were run on a 12% SDS-polyacrylamide gel and transferred onto a nitrocellulose membrane (Hybond-ECL, Amersham-Biosciences). The membranes were blocked with 5% (w/v) milk powder diluted in PBS-Tween 20 (0.05% v/v) (PBS-TM) and incubated for 1 h at room temperature with monoclonal mouse anti-myc 9E10 (Abcam Inc.) for VHH5-secreted or anti-E-tag antibodies for VHH2-anchored, both diluted 1/1,000 in PBS-TM, followed by HRP (horse radish peroxidase) labelled goat anti-mouse antibodies (1/1,000) (DAKO) for 1 h. The washed membrane was then developed with ECL Plus Western Blotting Kit (Amersham-Bioscience) according to the manufacturer's instructions.

### Enzyme-linked immunosorbent Assay

First, 96-well (Maxisorp) plates were coated with 10 μg/ml of recombinant major structural capsid protein (MCP, the VHH2 antigen) or recombinant receptor-binding protein (RBP, the VHH5 antigen) [5] and left overnight at 4°C. After washing with PBS containing 0.05% Tween 20 (PBS-T), dilutions of the concentrated supernatants from *L. paracasei *cultures secreting VHH5 as well as of the extract from cells anchoring VHH2, were added and incubated at room temperature for 1 h. Concentrated supernatants and cell extracts from cultures of *L. paracasei *containing only the vector pLP402 were used as negative controls. Purified VHH2 and VHH5 from *E. coli*, respectively with an E-tag and a c-*myc *tag [5], were used in standard curves to evaluate the amount of VHH produced by various *L. paracasei *transformants.

Plates were washed twice and a mouse anti-E-tag antibody (1/1,000) was added to the wells previously incubated with the extracts from *L. paracasei *VHH2-anchored. A mouse anti-myc antibody 9E10 (1/1,000) (Abcam Inc.) was added to the wells previously incubated with the purified VHH2 and VHH5 as well as supernatant containing VHH5 secreted by *L. paracasei *cells. After 1 h incubation at room temperature, plates were washed twice and an alkaline phosphatase conjugated rabbit anti-mouse antibody (1/1,000) (DAKO) was added to the plates. Following incubation for 1 h at room temperature, diethanolamine buffer (1 M, pH 10.0) containing 1 mg/ml of pNPP substrate (Sigma-Aldrich) was added to the wells. The absorbance was read at 405 nm in a Vmax Kinetic Microplate reader (Molecular Devices).

### Scanning electron microscopy (SEM)

A culture of *L. paracasei *anchoring VHH2 was washed in PBS and 100 μl (10^6 ^bacteria/ml) was mixed with 500 μl (5 × 10^10 ^pfu/ml) of the lactococcal phage p2 and left at room temperature for 1 h. The mixture was fixed in 2% glutaraldehyde diluted in 0.1 M sodium cacodylate and 0.1 M sucrose, and finally added onto a 0.1 mg/ml poly-L-lysine coated RC58 filter. After dehydration (70% ethanol 10 min, 95% ethanol 10 min, 99% ethanol 10 min), the samples were sputtered and analysed by SEM (JEOL JSM-820) at 15 kV.

### Phage inhibition assay

The protocol for this assay was adapted from the lactococcal 936-phage adsorption experiments of Geller *et al*. [24]. *L. paracasei *strains were grown to an optical density of 0.8 at 600 nm (OD_600_) in LCM-Man medium containing the appropriate antibiotic. Cells were harvested and the supernatant was filtered (0.45 μm) for immediate use. Cell pellets were suspended in 0.25 volume of LCM. Approximately 1,000 plaque-forming unit (pfu) of lactococcal phage p2 (30 μl) were mixed on ice with 10 μl of *L. paracasei *supernatant (about 5 ng VHH5), cells (4 × 10^6 ^bacteria) or LCM. After incubation of 4 h on ice, the mixture was centrifuged 5 min at 16,100 × *g *at 4°C. Phage titer was determined as followed in triplicate using 10 μl of the supernatant. Ten μl were added to 3 ml of GM17 supplemented with 0.75% agar and containing 100 μl of an overnight culture of the host strain *Lactococcus lactis *MG1363. The mixture was then poured onto a GM17 plate (1% agar), incubated overnight at 30°C, and the number of plaques were counted. The percentage of inhibition was calculated by dividing the titer of the phage with *L. paracasei *bacteria or supernatant by the phage titer in LCM. The quotient was subtracted from 1 and multiplied by 100. The experiment was repeated at least three times and the means were calculated for the pool of experiments. Because the *amy *promoter is repressed by lactose, co-culture of both strains in milk to test inhibition of phage infection was not tested with the present system.

## Competing interests

The author(s) declare that there are no competing interests.

## Authors' contributions

AH cloned the VHHs fragment, evaluated the expression and binding of the VHHs and wrote the first draft of the manuscript. DMT performed the phage inhibition assay. HdH and CTV provided the VHH genes, purified the recombinant phage proteins, and critically revised the manuscript. SM designed the protocol of phage inhibition and contributed to the writing of the manuscript. LH contributed to the development of experimental strategy as well as critically revising the manuscript. HM contributed to the development of experimental strategy as well as critically revising the manuscript. All co-authors read and approved the final manuscript.
